# Liver-HERO: hepatorenal syndrome-acute kidney injury (HRS-AKI) treatment with transjugular intrahepatic portosystemic shunt in patients with cirrhosis—a randomized controlled trial

**DOI:** 10.1186/s13063-023-07261-9

**Published:** 2023-04-05

**Authors:** Cristina Ripoll, Stephanie Platzer, Philipp Franken, Rene Aschenbach, Andreas Wienke, Ulrike Schuhmacher, Ulf Teichgräber, Andreas Stallmach, Jörg Steighardt, Alexander Zipprich, Liver-HERO Study Group, Philipp Reuken, Marina Reljic, Florian Bürckenmeyer, Kathleen Lange, Robin Greinert, Marco Damm, Christian Lange, Paul Jamme, Max Seidensticker, Moritz Wildgruber, Dominik Bettinger, Michael Schultheiss, Marco Berning, Stefan Sulk, Jens-Peter Kühn, Ralf-Thorsten Hoffmann, Christoph Radosa, Thomas Hofmockel, Tony Bruns, Theresa Wirtz, Philipp Bruners, Karel Caca, Andreas Wannhoff, Benjamin Massoumy, Katja Deterding, Jan Hinrichs, Kerstin Port, Jonel Trebicka, Michael Praktiknjo, Cornelius Engelmann, Andreas Drolz

**Affiliations:** 1 Internal Medicine IV, Jena University Hospital, Friedrich-Schiller-University Jena, Jena, Germany; 2 Center for Clinical Studies, Jena University Hospital, Friedrich-Schiller-University Jena, Jena, Germany; 3 Department of Radiology, Jena University Hospital, Friedrich-Schiller-University Jena, Jena, Germany; 4 Institute of Medical Epidemiology, Biometrics and Informatics, Martin-Luther-University Halle-Wittenberg, Halle, Germany; 5 Coordinating Center for Clinical Studies, University Medicine Halle (Saale), Martin-Luther-University Halle-Wittenberg, Halle, Germany

**Keywords:** Cirrhosis, Hepatorenal syndrome, Acute kidney injury, TIPS, Randomized controlled trial

## Abstract

**Background:**

Patients with cirrhosis and ascites (and portal hypertension) are at risk of developing acute kidney injury (AKI). Although many etiologies exist, hepatorenal AKI (HRS-AKI) remains a frequent and difficult-to-treat cause, with a very high mortality when left untreated. The standard of care is the use of terlipressin and albumin. This can lead to reversal of AKI, which is associated to survival. Nevertheless, only approximately half of the patients achieve this reversal and even after reversal patients remains at risk for new episodes of HRS-AKI. TIPS is accepted for use in patients with variceal bleeding and refractory ascites, which leads to a reduction in portal pressure. Although preliminary data suggest it may be useful in HRS-AKI, its use in this setting is controversial and caution is recommended given the fact that HRS-AKI is associated to cardiac alterations and acute-on-chronic liver failure (ACLF) which represent relative contraindications for transjugular intrahepatic portosystemic shunt (TIPS). In the last decades, with the new definition of renal failure in patients with cirrhosis, patients are identified at an earlier stage. These patients are less sick and therefore more likely to not have contraindications for TIPS. We hypothesize that TIPS could be superior to the standard of care in patients with HRS-AKI.

**Methods:**

This study is a prospective, multicenter, open, 1:1-randomized, controlled parallel-group trial. The main end-point is to compare the 12-month liver transplant-free survival in patients assigned to TIPS compared to the standard of care (terlipressin and albumin). Secondary end-point include reversal of HRS-AKI, health-related Quality of Life (HrQoL), and incidence of further decompensation among others. Once patients are diagnosed with HRS-AKI, they will be randomized to TIPS or Standard of Care (SOC). TIPS should be placed within 72 h. Until TIPS placement, TIPS patients will be treated with terlipressin and albumin. Once TIPS is placed, terlipressin and albumin should be weaned off according to the attending physician.

**Discussion:**

If the trial were to show a survival advantage for patients who undergo TIPS placement, this could be incorporated in routine clinical practice in the management of patients with HRS-AKI.

**Trial registration:**

Clinicaltrials.gov NCT05346393. Released to the public on 01 April 2022.

## Administrative information

Note: the numbers in curly brackets in this protocol refer to SPIRIT checklist item numbers. The order of the items has been modified to group similar items (see http://www.equator-network.org/reporting-guidelines/spirit-2013-statement-defining-standard-protocol-items-for-clinical-trials/).

**Table:** 

Title {1}	Liver-HERO: Hepatorenal Syndrome-acute kidney injury (HRS-AKI) treatment with transjugular intrahepatic portosystemic shunt in patients with cirrhosis. A randomized controlled trial
Trial registration {2a and 2b}.	Clinicaltrials.gov identifier: NCT05346393. Registered on 26^th^ of April 2022, https://clinicaltrials.gov/ct2/show/NCT05346393
Protocol version {3}	Version V01 of 03-AUG-2022
Funding {4}	German Research Fundation (Deutsche Forschungsgemeinschaft, DFG) Funding number: 431667134 Reference number: RI 3205/1–1
Author details {5a}	Cristina RIPOLL, Clinic for Internal Medicine IV, Jena University Hospital, Friedrich-Schiller-University Jena, Germany Stephanie PLATZER, Center for Clinical Studies, Jena University Hospital, Friedrich-Schiller-University Jena, Germany Philipp FRANKEN, Center for Clinical Studies, Jena University Hospital, Friedrich-Schiller-University Jena, Germany Rene ASCHENBACH, Department of Radiology, Jena University Hospital, Friedrich-Schiller-University Jena, Germany Ulrike SCHUHMACHER, Center for Clinical Studies, Jena University Hospital, Friedrich-Schiller-University Jena, Germany Andreas WIENKE, Institute of Medical Epidemiology, Biometrics and Informatics, Martin-Luther-University Halle-Wittenberg Andreas STALLMACH Clinic for Internal Medicine IV, Jena University Hospital, Friedrich-Schiller-University Jena, Germany Isabella SCHILLER, Center for Clinical Studies, Jena University Hospital, Friedrich-Schiller-University Jena, Germany Jörg STEIGHARDT, Coordinating Center for Clinical Studies, University Medicine Halle (Saale), Martin-Luther-University Halle-Wittenberg, Germany Alexander ZIPPRICH Clinic for Internal Medicine IV, Jena University Hospital, Friedrich-Schiller-University Jena, Germany Liver-HERO Study Group
Name and contact information for the trial sponsor {5b}	Friedrich-Schiller-University; sponsor representative Prof. Dr. Cristina Ripoll, Jena University Hospital, Clinic for Internal Medicine IV (Gastroenterology, Hepatology, Infectiology, Interdisciplinary Endoscopy), Am Klinikum 1, 07,747 Jena Tel: + 49 3641 9–32 42 29 Fax: + 49 3641 9–32 42 22 Cristina.Ripoll@med.uni-jena.de
Role of sponsor {5c}	The funder of the study (Deutsche Forschungsgemeinschaft, DFG) has no direct role in the design of the study, the writing of the protocol nor the decision to submit the report. The sponsor of the study is the Friedrich Schiller University, represented by the PI of the study, Prof. Cristina Ripoll, and who is responsible for every step of the trial.

## Introduction

### Background and rationale {6a}

Liver cirrhosis is a major cause of global health burden with 31 million disability-adjusted life years and 1 million deaths worldwide in 2010 [1]. Acute kidney injury (AKI) occurs in approximately 20% of hospitalized patients with cirrhosis [2, 3]. Approximately 2/3 of acute kidney injury are due to renal hypoperfusion, which in turn can be divided into prerenal AKI (responsive to administration of volume overload) or hepatorenal AKI (HRS-AKI) (unresponsive to the administration of volume overload) [2]. Hepatorenal syndrome (HRS) type 1 (now included in hepatorenal AKI) has a high mortality of almost 100% when left untreated [4] and is frequently part of multiorgan failure (acute on chronic liver failure). Among the etiologies of AKI in cirrhosis, hepatorenal AKI has the worst prognosis [5]. Treatment of HRS-AKI is based on the use of terlipressin and albumin, which leads to an improvement in renal function [6–9]. Patients with HRS reversal have reduced mortality [7, 8]. Despite the response to treatment, patients remain at risk for new episodes of HRS-AKI and death, so liver transplantation should be considered in these patients [10]. However, due to the limited organ availability and that many patients have contraindications to liver transplantation, this ideal possibility is feasible only in a few patients [11].

The transjugular intrahepatic portosystemic shunt (TIPS) is placed under radiological control and communicates the portal vein with a hepatic vein, leading to a reduction in portal pressure. Use of this shunt is part of the standard of care in patients with variceal bleeding and refractory ascites [12]. The use of transjugular intrahepatic portosystemic shunt (TIPS) in the context of HRS is rationally plausible as it reverses portal hypertension, one of the main drivers of HRS; nevertheless, this remains highly controversial [12]. On the one hand, it is well established that implantation of TIPS in patients with cirrhosis leads to beneficial effects for the kidney such as an improvement in renal blood flow [13], an improvement in renal autoregulation in response to the perfusion pressure [14], an improvement in the parameters of activation of the vasoactive system [15], an improvement in renal function [11, 15, 16], and a reduced incidence of HRS-AKI [17]. On the other hand, patients with HRS-AKI frequently have an acute-on-chronic liver failure with increased bilirubin and hepatic encephalopathy which may preclude TIPS implantation in these patients, due to high mortality [11, 12]. Furthermore, cardiac dysfunction due to cirrhotic cardiomyopathy is proposed to have a central role in the development of HRS-AKI [18]. TIPS leads to an increase in cardiac preload, so its placement should be evaluated on an individual basis, especially in the presence of diastolic dysfunction [19].

Two pilot studies have evaluated the use of TIPS in classical HRS type 1. One included only patients who had had a response to midodrine, octreotide, and albumin. From the 14 patients with HRS type 1, only 10 had a response to treatment, of which 5 could receive TIPS. After 6–30 months of follow-up, all patients with TIPS were alive (1 with liver transplantation), while in the other group, 3 had died (and 2 were alive with liver transplantation) [20]. A phase II study, evaluating TIPS in HRS (type 1 and 2) (*n* = 41) had a subgroup of patients with HRS type 1 (*n* = 21) [11]. Ten patients were excluded because of advanced liver failure, so 31 patients finally received TIPS (14 with HRS type 1). Survival improvement, both in the whole group and the subgroup with HRS type 1, was observed in the TIPS patients compared to those who were excluded from TIPS placement; however, the groups were by definition not comparable. Another retrospective study of patients with HRS type 1 with and without TIPS showed a better survival in those patients with TIPS; however, these patients had also a lower Child–Pugh score [21]. A recently published retrospective administrative database analysis has shown that patients with HRS who received a TIPS had decreased in-hospital mortality [adjusted OR 0.43 (95% CI 0.30–0.62]. Distinction between type 1 and type 2 HRS is not possible due to the study design [22]. The European Association for the Study of the Liver (EASL) guidelines [23] underline the lack of evidence to recommend TIPS placement in patients with HRS, while the American association for the study of liver diseases (AASLD) does not even mention this issue [24]. The German guidelines suggest that TIPS placement in these patients may be considered [25].

Furthermore, previous studies in the setting of another complication of cirrhosis, namely variceal bleeding [25–27], have shown that earlier TIPS placement leads to better outcomes due to a more preserved liver function when TIPS is placed. It is possible that an earlier TIPS implantation in patients with HRS-AKI stage 2 (instead of stage 3, which would mainly be patients with classical HRS type 1) could also have a better outcome. Indeed, previous indirect data as well as small pilot studies have suggested that TIPS may be helpful in selected patients with HRS-AKI. This study would be the first randomized controlled trial (RCT) to fill this gap of knowledge. If the trial were to confirm our hypothesis, TIPS placement would have a clear role in clinical practice since it could reduce mortality and morbidity and increase the quality of life in patients with cirrhosis and HRS-AKI.

## Objectives {7}

The primary objective of the study will be to evaluate if a transjugular intrahepatic portosystemic shunt (TIPS) implantation in patients with HRS-AKI improves survival. The secondary objectives will be to evaluate whether TIPS implantation in patients with HRS-AKI improves renal function and whether TIPS implantation in patients with HRS-AKI improves Health-related Quality of Life (HrQoL).

## Trial design {8}

Prospective, multicenter, open, 1:1-randomized, controlled parallel-group study.

*Framework/hypothesis*: the 12-month liver transplant-free survival in the experimental group (transjugular intrahepatic portosystemic shunt (TIPS)) is superior to the control group (standard of care; terlipressin and albumin).

## Methods: participants, interventions, and outcomes

### Study setting {9}

German national study with 10–15 participating study sites (academic hospitals and community clinics). List of participating sites can be obtained from continuously updated entries found in the registry on clinicaltrials.gov [28].

### Eligibility criteria {10}

#### Inclusion criteria

1. Patients with cirrhosis confirmed by histology or liver stiffness or with unequivocal signs in ultrasound, endoscopy, and/or blood tests
2. Clinically evident ascites due to portal hypertension (serum-ascites-albumin-gradient (SAAG) > 1.1 g/dL)
3. HRS-AKI stages 2 or 3
4. Planned vasoactive treatment for the management of HRS, as defined by the administration of terlipressin + albumin
5. Age: ≥ 18 to ≤ 75 years old at the time of consent
6. ECOG (Eastern Cooperative Oncology Group) score < 4 prior to hospital admission
7. Subject has been informed of the nature of the study, is willing to comply with all required follow-up evaluations within the defined follow-up visit windows, and has signed an Ethics Committee (EC) approved consent form.
8. Female subjects of childbearing potential have a negative pregnancy test ≤ 7 days before the procedure and are willing to use a reliable method of birth control for the duration of study participation. Female subjects will be exempted from this requirement in case they are sterile, infertile, or have been post-menopausal for at least 12 months (no menses). A contraceptive method with a pearl index below 1% is assumed to be effective.

#### Exclusion criteria

1. Patients with signs of intrinsic renal disease as defined by proteinuria (> 500 mg per day), microhematuria (> 50 red blood cells (RBC) per high power field), or signs of chronic renal disease on ultrasound.
2. Recent or current use of nephrotoxic drugs (nonsteroidal anti-inflammatory drugs (NSAIDs), aminoglycosides, or iodinated contrast medium) in the previous 72 h before AKI diagnosis
3. Improvement of renal function after 2 days of diuretic removal and plasma volume expansion with albumin 1 gr/kg
4. Uncontrolled shock
5. Patients with uncontrolled infection (defined by a 20% increase in inflammatory parameters (C-reactive protein (CRP), leucocytes or insufficient decrease of polymorphonuclear leukocytes (PMN) in ascitic fluid < 25% from baseline in the case of spontaneous bacterial peritonitis (SBP))) despite 48 h of antibiotic treatment.
6. Patients with cardiac cirrhosis as defined by the development of cirrhosis in a patient with chronic heart failure due to a primary cardiac disease (ischemic cardiomyopathy, hypertensive cardiomyopathy, etc.)
7. Patients with contraindications to TIPS placement (bilirubin > 5 mg/dL, recurrent hepatic encephalopathy)
8. Patients with cavernous portal vein thrombosis, splenic vein thrombosis, or mesenteric vein thrombosis
9. Patients with clinically significant cardiac disease (NYHA ≥ II)
10. Patients with diastolic dysfunction grade 3
11. Patients with a reduced systolic function with an ejection fraction ≤ 50%
12. Patients with an acute variceal bleeding at the time of screening who have indication for pre-emptive TIPS and/or terlipressin
13. Patients with refractory ascites as defined by the International Ascites Club (IAC) [29] (< 800 g weight loss over 4 days in patients on low salt diet and high dose diuretics (spironolactone 400 mg/day and furosemide * 160 mg/day), or lower dose of diuretics with complications secondary to the use of diuretics such as hyponatremia, renal failure, hepatic encephalopathy. *Equivalent dose of torasemide 40 mg/day)
14. Patients with hepatocellular carcinoma outside of the Milan criteria
15. Patients with hepatocellular carcinoma within the Milan criteria in whom the tumor is located in the puncture tract.
16. Patients with benign liver tumors (except regenerative nodules) which are located in the puncture tract.
17. Patients with other comorbidities that lead to an estimated life expectancy of under 1 year.
18. Patients with respiratory insufficiency which requires mechanical ventilation
19. Patients with circulatory failure which requires the administration of catecholamines
20. Patients receiving renal replacement therapy
21. The subject is currently enrolled in another investigational device or drug trial
22. Patients with pregnancy or lactation

#### Eligibility criteria for participating sites and investigators

The participating site must be equipped with the appropriate resources to fulfill the clinical study requirements as described in the protocol. All sites are centers with a multidisciplinary team (including hepatologists, interventional radiologists (in the case the TIPS are placed by them), intensive care and general surgery with expertise in liver surgery and ideally liver transplantation) and experience in the treatment of patients with decompensated cirrhosis. It is known that the adjusted mortality rate after TIPS placement decreased in centers which placed more than 20 TIPS per year [30]. The median number of TIPS placed in German centers with expertise in TIPS placement was 28/year [31]. The procedure should be done by an interventional radiologist or a gastroenterologist/hepatologist who has experience in this procedure having performed at least 5 TIPS under supervision beforehand and at least 15 TIPS without supervision.

### Who will take informed consent? {26a}

Prior to inclusion and in accordance with the Declaration of Helsinki and national regulations, patients must undergo the consent process. During the consent process, the investigator or his/her designee must fully inform the patient about all relevant study details including potential risks and benefits of participation. Written informed consent is prerequisite for inclusion into the study.

### Additional consent provisions for collection and use of participant data and biological specimens {26b}

Biological specimens are collected during the study. Blood, ascites (at baseline), and urine samples are obligate; stool samples are optional. The participants are asked for additional consent for stool sampling. These samples will be used for the present study and future ancillary studies.

### Interventions

#### Explanation for the choice of comparators {6b}

The control group will be selected by the same inclusion and exclusion criteria as the intervention group and undergo the usual standard of care treatment with terlipressin and albumin. Thus, the outcome in this group will reflect the status quo in therapy of patients suffering from HRS-AKI. The patients in the treatment group are also treated with standard of care and then with a TIPS. Consequently, any difference in the outcome between both groups should be attributable to the TIPS. Due to the nature of the intervention, a double-blind design is not possible.

#### Intervention description {11a}

##### Administration of terlipressin and albumin (SOC) in both groups

Once the diagnosis of HRS-AKI is established, the standard of care will be administered according to actual clinical practice guidelines [23] following the judgment of the attending physician (see below). To bridge the time gap until the TIPS placement (scheduling for radiological intervention; no longer than 72 h), patients in the interventional group will also receive standard of care until TIPS is placed. After TIPS placement the medication is to be weaned and discontinued.

All patients with AKI will receive according to clinical practice guidelines albumin infusion 1 g/kg infusion (up to a maximum of 100 g) for 2 days [23]. Lack of response to volume overload is defined as improvement of serum creatinine < 25% from the peak value. This administration of albumin is required for the diagnosis of AKI-HRS and is prior to the inclusion in the study.

Terlipressin will be started in continuous perfusion (2–4 mg per day) and will be increased in a stepwise fashion every 2–3 days in the case of non-response of serum creatinine (decrease in serum creatinine < 25% from peak value). The maximum dose of terlipressin is 12 mg/day. Albumin will be given at a dose between 20 and 40 mg per day and adjusted according to the volume status of the patient. Treatment will be maintained until achieving a full response (defined by regression of serum creatinine to 26.5 µmol/L from baseline value) or a maximum of 14 days.

Special attention will be given to volume overload. This will include daily physical examination including pulmonary auscultation and if considered necessary by the managing physician chest X-ray or measurement of central venous pressure or inferior vena cava diameter on ultrasound. If clinical suspicion of mild volume overload albumin will be reduced, if clinical suspicion of severe volume overload albumin will be temporarily suspended. Veno-venous hemodialysis will be initiated according to the decision of the managing physician. It is recommended that patients who require veno-venous hemodialysis do not receive further treatment with terlipressin + / − albumin. The patient will remain in the study for the intention to treat analysis regarding the main end-point.

##### TIPS procedure/interventional group

Placement of TIPS is an interventional radiological procedure. A central access over the right jugular vein is obtained. Through this central access, the right hepatic vein is catheterized and then a needle is introduced in the hepatic vein. Under fluoroscopic and sonographic guidance, the needle is introduced through the liver parenchyma to the (right) portal vein branch. Once in the portal vein, the puncture tract (between the hepatic vein and the portal vein) is dilated with a balloon and an 8–10-mm controlled-expansion ePTFE (expanded PTFE) covered stent-graft is placed. The TIPS should be placed initially with an 8-mm diameter.

A second measurement of the portal pressure gradient should be done after a maximum of 72 h. In this procedure, the portal pressure gradient will be calculated as the difference between the pressure in the main portal vein and in the vena cava inferior at the junction with the hepatic veins. If terlipressin is still being administered by the second measurement, this medication should be paused at least for 6 h before the measurement.

The aim is to reduce the pressure to 8–10 mmHg, depending on the clinical characteristics of the patient. In the case of a very high (> 24 mmHg) or very low (< 15 mmHg) initial gradient, the aim would be to reduce the pressure from 25 to 50% [29]. All measurements of pressure must be registered in paper or digital format for at least 15 s.

A routine TIPS requires approximately 15–20 min of X-ray fluoroscopy. Different measures (such as reducing the field of exposure, reducing the rate of fluoroscopy) are undertaken to reduce exposure. A second measurement of the portal pressure gradient requires on average approximately 2 min of X-ray fluoroscopy. During the procedure, use of contrast medium should be minimized. Management of patients after TIPS should be done according to local standards; anticoagulation or anti-aggregation is not recommended taking into account the increased risk of these treatments in the context of AKI.

#### Criteria for discontinuing or modifying allocated interventions {11b}

There are two contexts in which a patient may modify the allocated intervention. These are named in the context of the trial switchers.

T-to-C-switcher: patients, randomized in the TIPS group (T), but who are not in the constitution for this procedure or develop contraindications for TIPS procedure between randomization and day of planned TIPS placement (max 72 h), respectively and drop into standard of care treatment (Control, C).

C-to-T-switcher: patients, who are initially assigned to standard of care (C) but develop complications of cirrhosis in which TIPS placement is indicated during follow-up and hence drop in TIPS treatment (T).

#### Strategies to improve adherence to interventions {11c}

Given the dynamic nature of AKI-HRS, no specific measures can be applied to avoid T-to-C switchers, besides strict application of the inclusion and exclusion criteria. In order to avoid C-to-T switchers, specific criteria for the placement of TIPS (control group) or revision of TIPS (intervention group) have been specified. TIPS placement and TIPS revision are indicated in the case of development of:

- Massive bleeding requiring placement of an ELLA Danis stent or Sengstaken-Blakemore or Linton balloon (emergency TIPS)
- Esophageal variceal bleeding (in patients with Child–Pugh B 7–9 points and active bleeding at endoscopy or patients with Child–Pugh C 10–13 points) (pre-emptive TIPS)
- Recurrent esophageal bleeding despite adequate secondary prophylaxis with beta-blockers and endoscopic band ligation
- Refractory ascites as defined by the IAC (see Ch. 14.3) [32]
- Recurrent ascites as defined by the need of 3 large paracenteses within 3 months (in a stable situation). Only paracentesis that takes place in a stable situation, namely without concurrent infection, without AKI, without bleeding, etc., will be considered [23].

If a patient randomized into the control group develops an indication for TIPS (except emergency TIPS or pre-emptive TIPS) during follow-up, the treating study physician should consult the Principal Coordinating Investigator to discuss the indication before TIPS implantation.

#### Relevant concomitant care permitted or prohibited during the trial {11d}

All indicated standard of care therapy is permitted during the trial. Placement of TIPS or revision of TIPS during follow-up is permitted with strict adherence to the accepted criteria (see Strategies to improve adherence to interventions {11c} section).

#### Provisions for post-trial care {30}

Once the patient finalizes the follow-up in the study, he/she will return to routine clinical follow-up in the outpatient clinic every 3–6 months depending on the clinical condition including screening ultrasound every 6 months when indicated.

All patients participating in the trial will have insurance coverage by the sponsor which is in line with the applicable law and regulations, covering in its terms and provisions, its legal liability for injuries caused to participating persons arising out of this research performed strictly in accordance with scientific protocol as well as applicable law and professional standards. Insurance includes accidents on the way from the patient’s home to the study site and back; Insurance: HDI Global SE, insurance number: NEV050158A.

### Outcomes {12}

The primary endpoint is the 12-month liver transplant-free survival. This endpoint has been recently recommended as the primary endpoint to be reported in trials in decompensated cirrhosis [33]. This endpoint is a hard clinical endpoint, which is evidently patient-relevant.

Secondary endpoints include:

- Reversal of HRS-AKI at 3 and 12 months (vs baseline), defined as the return of serum creatinine level within 0.3 mg/dl (26 µmol/L).
- Partial response to treatment at 3 and 12 months (vs baseline), defined as reduction of at least one AKI stage with decrease of serum creatinine to ≥ 0.3 mg/dl (26 µmol/L) above the baseline value.
- 3-month liver transplant-free survival
- In-hospital, 28-day, and 90-day survival
- Development of cirrhosis-associated complications (further decompensation) during follow-up: overt hepatic encephalopathy, refractory or recurrent ascites, dilutional hyponatremia, new AKI-HRS, variceal bleeding
- Requirement of TIPS implantation or TIPS revision during follow-up. The accepted indications for TIPS implantation/revision are
  ◦ Pre-emptive TIPS for variceal bleeding in patients with Child–Pugh C cirrhosis (≤ 13 points) or Child–Pugh B > 7 points cirrhosis with active bleeding at endoscopy
  ◦ Recurrence of variceal bleeding in Child–Pugh A or B 7 points patients or Child–Pugh B patients without active bleeding at endoscopy despite adequate secondary prophylaxis with beta-blockers and endoscopic band ligation
  ◦ Refractory ascites—this diagnosis can only be established if the patient is in a stable situation without complications such as bleeding and infection
  ◦ Recurrent ascites—this diagnosis can only be established if the patient is in a stable situation without complications such as bleeding and infection
  ◦ Additionally, TIPS revision can be undertaken if there is a clinical suspicion of TIPS dysfunction
- Development of acute on chronic liver failure (ACLF) during follow-up
- Length of in-hospital-stay
- Relative changes in HrQoL (as measured by SF36 and Chronic Liver Disease Questionnaire (CLDQ)) at 3 and 12 months (vs. baseline)
- The incidence of adverse events in the TIPS patients compared to standard of care (with specific focus on ACLF and heart failure)
- Need for renal replacement therapy
- Recurrence of HRS-AKI after treatment at 3 and 12 months
- Impact of the presence of intrinsic nephropathy as assessed by cystatin C and Urine neutrophil gelatinase-associated lipocalin (UnGAL) on outcomes
- Association of pathophysiological mechanisms of cirrhosis with outcomes (in further studies)

### Participant timeline {13}


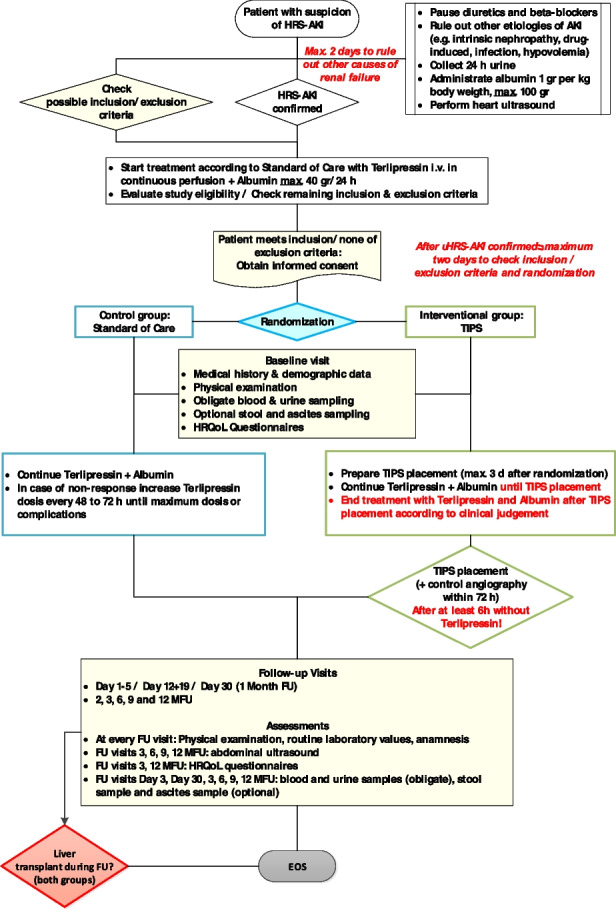


**Table:** 

	Pre-screening **within **SoC/confirmation of HRS-AKI diagnosis	Screening/baseline	FU days 1–5	FU days 12 and 19	FU day 30 (1 MFU)	FU 2 months (2 MFU)	FU 3 months (3 MFU)	FU 6 and 9 months (6 MFU, 9 MFU)	FU 12 months (12 MFU)
Medical history/anamnesis^a^	X	X							
Demographic data		X							
Physical examination^b^	X	X	X	X	X	X	X	X	X
Routine laboratory examination^c^	X	X	X	X	X	X	X	X	X
24-h urine	X				X				
uETG/alcohol consumption		X		X	X	X	X	X	X
Echocardiography	X								
Abdominal ultrasound	X						X	X	X
Diagnostic paracentesis	X								
Chest-X-ray	X								
Inclusion/exclusion criteria		X							
ICF		X							
Randomization		X							
Blood and urine sampling		X	X (D3)		X		X	X	X
Optional ascites sampling		X	X (D3)		X		X	X	X
Optional stool sampling		X					X	X	X
Terlipressin/albumin treatment^d^		X	#						
TIPS		X	Angio^e^						
HrQoL Questionnaires (SF-36, CLDQ)		X					X		X
AE/SAEs		X							
Length of in-hospital stay		X							
Liver transplantation performed		X							
Requirement of renal replacement therapy		X							

^a^Medical history/anamnesis includes questions on the occurrence of HCC, medication intake, actual status of HRS-AKI/ascites (previous and actual therapies/measures, decompensation), and intake of low-salt diet.

^b^Physical examination includes evaluation of orientation (time, person, place) and presence of flapping tremor and evaluation of skin, heart, and lung auscultation; abdominal physical examination; and presence of lower limb edema.

^c^Laboratory values (assessed by local lab): (1) in blood: creatinine, sodium, potassium, ALAT, ASAT, GGT, AP, bilirubin, albumin, CRP, INR, hemoglobin, hematocrit, leukocytes, platelets; (2) in ascites/pleural effusion: albumin, leukocytes, neutrophils, glucose, LDH, bilirubin.

^d^For test arm until TIPS placement and then will be progressively discontinued according to the managing physician’s decision. For SoC arm, the treatment will be given beyond baseline (#) and continued according to the Clinical Practice Guidelines and the managing physician’s decision.

^e^Control angiography within 72 h after TIPS placement (measurement should be performed after pausing Terlipressin for at least 6 h).

### Sample size {14}

The sample size estimation is based on the primary outcome transplant-free survival (LTX-OS) and two-sided log-rank test. Previous data suggest that type 1 HRS who received TIPS had a 64% survival at 3 months, with a 20% 1-year survival [10]. In the treatment arms of the RCT in HRS evaluating the combination of terlipressin and albumin, the 3-month transplant-free survival rate was 26% [6–9].

Because of the change in the definition of HRS, the patients in the present study will be in a slightly better shape, so an estimated 3-month survival rate of 50% (exponential parameter 0.231) in the control and 70% (exponential parameter 0.1189) in the experimental group (resulting in a hazard ratio HR = 0.51) are expected. With a significance level of 5%, power of 90%, recruitment period of 24 months, individual follow-up of 12 months, and dropout rate of 10% (exponential parameter 0.0088), 56 patients are needed in each group.

Due to the expected additional T-to-C-switcher (patients, randomized in the TIPS-group, but who are not in the constitution for this procedure) between randomization and TIPS procedure, 62 patients will be randomized to each group (124 patients overall). Although there are no data regarding this, from clinical experience we believe that this will happen in approximately 10% of patients. As the adjusted Cox regression analysis is more powerful than the log-rank test, this sample size calculation is considered to be conservative. Furthermore, patients who are initially assigned to the C group may develop during follow-up complications of cirrhosis in which TIPS placement is indicated. There is no clear-cut data about the number of patients in whom this will occur and hence drop in TIPS treatment (C-to-T-switcher). Assuming that this number will be low, a relatively high power of 90% was chosen to account for possible switchers, and the sample size is calculated without this information.

### Recruitment {15}

It is intended to include 124 patients at approximately 10–15 sites in Germany within 24 months which is less than 1 patient per month per site. There is no limit to the number of subjects randomized at any individual clinical site. AKI-HRS typically requires hospitalization for its management. These patients are typically hospitalized in hepatology wards or intensive care units. Therefore, only in-patients will be considered for inclusion. All patients fulfilling the inclusion and not fulfilling the exclusion criteria will be considered for the study. Educational measures will be taken to increase awareness in referral hospitals. It is estimated that 2 years will be required to include the patients.

## Assignment of interventions: allocation

### Sequence generation {16a}

Patients will be randomized in a 1:1 ratio to the TIPS or the SOC group. The randomization will be stratified according to the AKI stage (II or III) and sites. Randomization will be restricted by randomly varying block size and stratification by center. A computer-generated randomization list will be prepared by an independent statistician not involved in enrolment and analyses using the software nQuery Advisor. Randomization of a patient will be done by the site via an online tool of ZKS Jena (PaRANDies) to ensure that group assignment is unbiased and concealed from patients and investigator staff.

### Concealment mechanism {16b}

The randomization is done via an online tool (see Sequence generation {16a} section), which permits concealment of the randomization sequence.

### Implementation {16c}

Allocation sequence will be generated by an independent statistician who is not involved in enrolment and analyses.

## Assignment of interventions: blinding

### Who will be blinded {17a}

The study is open. Given the nature of the intervention, blinding of the attending physicians is not ethically feasible. Blinding of the data analyst is not possible.

### Procedure for unblinding if needed {17b}

Not applicable. The design is open-label so unblinding will not occur.

## Data collection and management

### Plans for assessment and collection of outcomes {18a}

#### Health-related Quality of Life assessment

Health-related Quality of Life (HrQoL) will be assessed using the two questionnaires SF-36 [34–39] and CLDQ [40, 41] at baseline and FU visits at 3 and 12 months. Both patient-reported outcome questionnaires are globally established and widely used in clinical research. The SF-36 will give information on overall patient health, while the CLDQ will provide information on disease-specific patient health.

### Plans to promote participant retention and complete follow-up {18b}

Participants will only be included if they are aware of the follow-up and agree to comply with the follow-up visit schedule. Upon discharge, they receive the visit schedule. At each follow-up, the site will try to contact the participant by telephone (up to three times) and, if necessary, by letter (one time) before participant is classified as lost to follow-up. If participants are unable or unwilling to on-site visits, they will be asked to answer questions by phone. If participants decline further phone contact, they will be asked to authorize the release of medical information concerning safety events by their general practitioner or family members. In case all attempts fail, the site may ask the participant if he/she is willing to accept a phone call at the end of the study.

### Data management {19}

Medical data will be entered by means of an online data collection system and transmitted directly to the central data management (center for clinical studies [ZKS] Jena). Transfer of patient-related medical data will be carried out pseudonymized. No features will be transferred that enable immediate identification of specific participants by the data management. Data entry, processing, and evaluation will comply with the provisions of the German Federal Data Protection Act (BDSG) and the EU General Data Protection Regulation (EU-GPDR) Records and documents related to the clinical trial must be kept for at least 15 years.

### Confidentiality {27}

Investigators and study staff will keep all information on participants in strict confidence. Appropriate local data legislation will be applied in full. A confidential log of the names of all study patients with the identification code assigned to each patient at the time of enrolment in the clinical study will be filed at each site. With this list, the identity of each patient can be revealed (key list for pseudonymization). The list must be kept confidential and must be maintained and stored exclusively at the study site in the investigator site file (ISF). The personal data collected and generated in the course of the study are processed and stored at the sponsor site (Jena University Hospital) solely based on the patient identification number in order to maintain the pseudonymization. The data managers of the ZKS Jena will have access to all clinical trial data. These persons are sworn to secrecy. The data will be protected against unauthorized access. The monitor, safety manager, and trial statistician will also have access to several clinical trial data as well as study leaders and statisticians for any substudies including future meta-analyses.

### Plans for collection, laboratory evaluation, and storage of biological specimens for genetic or molecular analysis in this trial/future use {33}

Blood and urine and optional stool samples for the central laboratory will be obtained at baseline, 3, 6, 9, and 12 months. These will be obtained in order to evaluate in further research the pathophysiological mechanisms involved in the development of HRS-AKI such as the activation of vasoactive parameters (Renin, Aldosterone, Noradrenalin) and bacterial translocation (IL-6, Endocab, LBP). An initial planned exploratory subanalysis will be performed according to blood and urine markers of functional or organic renal impairment (Cystatin C and uNGAL). Stool samples will be optionally collected for future exploratory analysis regarding the influence of the gut microbiome on outcomes. Embedded future substudies for research on HRS will have their specific protocol and will obtain specific approval from the ethic committee separately.

## Statistical methods

### Statistical methods for primary and secondary outcomes {20a}

#### Populations for analysis

The primary analysis data set is the intention-to-treat population. This data set contains all the patients who have been enrolled in the clinical trial and randomized.

The secondary analysis data set is derived from the per-protocol population. This data set includes all patients who have been treated according to the protocol during the whole duration of the study and reached a defined endpoint.

The tertiary analysis data set (safety-population) contains all the patients who have received the trial procedure.

#### Methods of analysis

All compiled data will be analyzed at least in a descriptive manner. Including count of compiled data and missings, mean, standard deviation, minimum, quartiles, and maximum for metric and frequency analysis for ordinal and categorical data.

Demography: age, sex, size, weight, racial background.

We hypothesize that the 12-month liver transplant-free survival is higher in patients in the interventional group. We will test the hypothesis

$$\mathrm H0:\;\mathrm{HR}\;=\;1.0\;\mathrm{vs}.\;\mathrm{HA}:\;\mathrm{HR}\;\neq\;1.0.$$

The primary endpoint will be analyzed based on the intention to treat principle using a Cox regression adjusted for the AKI stage.

We will perform a competing-risk analysis in addition to the primary endpoint with the competing events of death and liver transplantation. As secondary outcomes reversal of HRS-AKI (vs baseline), partial response to treatment (vs baseline), need of renal replacement therapy, and recurrence of HRS-AKI at 3 and 12 months will be assessed by logistic regression adjusted for AKI stage. In-hospital, 28-day, and 90-day survival will be assessed by logistic regression adjusted for AKI stage. Changes in HrQoL at 3 and 12 months with respect to study baseline will be compared between groups by linear regression adjusted for AKI stage. Three-month liver transplant-free survival will be analyzed using a Cox regression adjusted for AKI stage. Development of further decompensations and length of in-hospital stay will be analyzed descriptively. Results will be interpreted in an exploratory manner.

The number of AEs and SAEs in each group with special attention on the development of ischemic hepatitis, acute on chronic liver failure, hepatic encephalopathy, and signs of heart failure will be analyzed. Laboratory assessments including sodium, potassium, ALAT, ASAT, GGT, AP, bilirubin, albumin, and INR will be analyzed descriptively as safety parameters.

### Interim analyses {21b}

No interim analyses are planned.

### Methods for additional analyses (e.g., subgroup analyses) {20b}

The primary endpoint will be analyzed in a secondary analysis based on the intention to treat principle using a Cox regression (with centers, presence/absence of intrinsic renal damage as determined by plasma and urine biomarkers, etiology of the underlying liver disease (alcoholic versus non-alcoholic) and impact of the presence of intrinsic nephropathy as assessed by cystatin C and UnGAL as fixed effects) adjusted for AKI stage. These effects will be checked with BIC (Bayesian information criterion). In case of significance, results will be interpreted in an exploratory manner.

### Methods in analysis to handle protocol non-adherence and any statistical methods to handle missing data {20c}

Drop-outs will be dealt with as independent right censored in the primary analysis. All patients will be analyzed in their randomization group. Because both switcher groups (definition in Criteria for discontinuing or modifying allocated interventions {11b}) are not random inside their groups, a per-protocol analysis will be performed as sensitivity analysis of the primary outcome. Drop-outs and C-to-T-switcher will be dealt with as independent right censored in this analysis without T-to-C-switchers.

Additional both analyses will be performed with requirement of TIPS implantation/revision in the follow-up as third endpoint.

#### Missed visits

It is possible that patients miss to attend one or more visits but can be followed up until the regular end of the study at the 12-month FU. The number of missed visits at the respective time points will be given in the CONSORT 2010 flow diagram.

### Plans to give access to the full protocol, participant-level data, and statistical code {31c}

Public access can be given to the full protocol, anonymized participant-level dataset (upon reasonable demand), and statistical code (upon demand), with the publication of the manuscript reporting the results of the trial according to the requirements of the journal.

## Oversight and monitoring

### Composition of the coordinating center and trial steering committee {5d}

The coordinating center consists of the principal coordinating investigator (PCI) (CR, sponsor representative, Friedrich-Schiller-University Jena, Germany), the co-principal coordinating investigator (AZ), the trial manager (SP), the statistician (PF), the data and safety management team (center for clinical studies Jena (ZKS Jena), Jena University Hospital, Germany), and the monitoring team (Coordinating Center for Clinical Studies Halle (KKS Halle), Martin Luther University Halle, Germany). The principal coordinating investigator and co-principal coordinating investigator together with the independent data and safety monitoring board (DSMB) assume the role of the trial steering committee.

### Composition of the data monitoring committee, its role, and reporting structure {21a}

The data and safety monitoring board (DSMB) is established in order to monitor the safety of participants. The DSMB consists of two independent physicians and a statistician with pertinent experience who may review study information during the conduct of the trial. Their major responsibility is to make recommendations on further study conduct. Any premature termination or suspension of the trial must be discussed with the DSMB. The DSMB will review a safety event dossier, provided by the sponsor for all reported cases of severe adverse events and death.

### Adverse event reporting and harms {22}

Adverse events are any untoward medical occurrences, unintended diseases or injuries, or any untoward clinical signs including abnormal laboratory findings in participants, users, or other persons in the context of this study, whether or not related to the investigational or control device and procedure. For users and other persons, adverse events are restricted to related adverse events. All adverse events must be specified in the study adverse event case report form. Severity and putative relationship to study devices or procedures should be noted. Investigational sites are responsible for adverse event reporting to the sponsor. Device complaints have to be reported directly to the manufacturer. Adverse device effects will be reported to the sponsor (ZKS Jena) quarterly.

Serious adverse events are any untoward events that occur during this study, which lead or possibly might lead, directly, or indirectly to death or serious deterioration in the state of health, life-threatening illness, injury, or permanent impairment of a body structure or a body function including chronic diseases, or prolonged hospitalization, or medical or surgical interventions to prevent life-threatening illness or injury, or permanent impairment to a body structure or a body function of a participant, user, or other persons whether or not related to the investigational or control device and procedure. For users and other persons, serious adverse events are restricted to related serious adverse events. In the event of severe adverse events, investigational sites must immediately deliver a report to the sponsor (center for clinical studies [ZKS] Jena, via fax within 24 h of knowledge). Any required follow-up information must be provided as soon as possible. All severe adverse events that are still ongoing at 12 months have to be followed up until resolved or until investigator confirms that no further improvement or deterioration is expected. The same applies to adverse events of special interest (AESIs) if not already fulfilling the definition of a SAE. AESIs are adverse events defined as especially critical for patient safety within this study: ischemic hepatitis, ACLF, heart failure, hepatic encephalopathy, and development of post-contrast (PC)-AKI.

Incidents of any medical device that have occurred in Germany, irrespective of a clinical study, have to be reported to the competent authority by the device user. The sponsor will send a quarterly report with the cumulative severe adverse event assessment to the Federal Institute for Drugs and Medical Devices (BfArM) and ethical committees involved as required.

The trial may be terminated prematurely if the DSMB raise concerns about the safety of the medical device.

### Frequency and plans for auditing trial conduct {23}

Inspections of the ongoing or already completed study can be carried out by the respective competent authorities in accordance with the applicable legislation. In addition, sponsor’s representatives can conduct monitoring and audits at participating institutions at any time as part of quality assurance.

Monitoring includes initiation, regular on-site, and close-out visits. Monitoring will be carried out by appropriately trained clinical research associates according to the standard operating instructions of the responsible clinical research organization (KKS Halle, Martin-Luther-University Halle-Wittenberg, Germany). Frequency of regular and interim visits will depend on the study monitoring plan, recruitment rate, study compliance, and findings from previous visits. Principal investigators or the institutions involved will give the monitor/auditor access to all documents necessary for review.

### Plans for communicating important protocol amendments to relevant parties (e.g., trial participants, ethical committees) {25}

All substantial changes in the study protocol or other documents required for approval will be advertised to the respective competent authorities and the responsible ethics committee according the current valid legislation at the respective time point. Implementation of a substantial amendment can only occur after formal approval of the responsible ethics committee and regulatory authority.

### Dissemination plans {31a}

Progress reports and a final report at study termination will be prepared under the responsibility of the sponsor and provided to the reviewing ethics committees as required by local regulations. Publication policy of this study has been negotiated and specified in contractual obligations and agreements between involved centers.

The results of the study will be presented in national and international congresses. The results will be published in peer-reviewed journals. Once the results are published, these will be disseminated among the members of the gastroenterology and hepatology community by means of inclusion in national and international clinical practice guidelines.

## Discussion

There are several aspects that have been identified as possible practical and operational issues in the study, which have been partially previously discussed. Firstly, the present definition of AKI requires paying special attention to variations in renal function even within the normal range. Although this definition of AKI has been in place for several years, it still has not permeated completely to clinical practice. This could lead to a reduction in the identification of patients as possible candidates for the study and could maybe impact recruitment. In order to tackle this issue, the PI will undertake educational measures to increase the awareness of the study teams.

The other main operational issue is the implantation of TIPS during follow-up in the standard of care group since frequently the strict definitions of refractory ascites and recurrent ascites are not fulfilled. In order to approach this issue, educational measures on behalf of the PI will take place to increase awareness of the need to follow the established definitions of refractory ascites and recurrent ascites. If the patient fulfills the accepted definitions of refractory ascites and recurrent ascites, TIPS placement is permitted. The cases that fulfill this indication should be discussed with the PI on a case-by-case basis before TIPS implantation. Similarly, if the patient fulfills the bleeding indications for TIPS (pre-emptive TIPS, salvage TIPS, rescue TIPS), TIPS placement is permitted during follow-up without discussion with the PI. The placement of TIPS during follow-up in the SOC will be specifically monitored by the study steering committee.

## Trial status

Protocol version V02 of 24-NOV-2022

Start of recruitment was Q1/2023, Last Patient In is planned for Q4/2024. Last Patient Out with completed 12-month follow-up is planned for Q4/2025.

## Data Availability

The PI, co-PI, and ZKS will have access to the final trial dataset. Members of the Liver-HERO Study Group may propose ancillary studies. The written proposal of these studies will be evaluated by the steering committee. In the case the study is accepted, the PI of the ancillary study will have access to the final trial dataset.

## Abbreviations

AASLD: American Association for the Study of Liver Diseases
ACLF: Acute-on-chronic liver failure
AE: Adverse event
AKI: Acute kidney injury
BfArM: Federal Institute for Drugs and Medical Devices (Bundesinstitut für Arzneimittel und Medizinprodukte)
CLDQ: Chronic Liver Disease Questionnaire
DFG: Deutsche Forschungsgemeinschaft (German Research Fundation)
EASL: European Association for the Study of the Liver
ECOG: Eastern Cooperative Oncology Group
FU: Follow-up
HrQoL: Health-related quality of life
HRS-AKI: Hepatorenal AKI
IAC: International Ascites Club
ICH-GCP: International Conference on Harmonization – Good Clinical Practice
KKS: Coordinating Center for Clinical Studies
LPB: Lipopolysaccharide binding protein
LTX-OS: Liver-transplant-free overall survival
MFU: Month follow-up
NSAID: Nonsteroidal anti-inflammatory drugs
NYHA: New York Heart Association
PC: Post-contrast
PMN: Polymorphonuclear leukocytes
PTFE: Polytetrafluoroethylene
RBC: Red blood cells
SAAG: Serum ascites albumin gradient
SAE: Serious adverse event
SBP: Spontaneous bacterial peritonitis
SOC: Standard of care
TIPS: Transjugular intrahepatic portosystemic shunt
UnGAL: Urine neutrophil gelatinase-associated lipocalin
ZKS: Center for Clinical Studies
